# Research hotspots and new trends in the impact of resistance training on aging, bibliometric and visual analysis based on CiteSpace and VOSviewer

**DOI:** 10.3389/fpubh.2023.1133972

**Published:** 2023-06-02

**Authors:** Junmin Mi, Litao Zhang, Wei Sun, Zhen Wang, Pengbo Yang, Jiachen Zhang, Yani Zhang

**Affiliations:** ^1^Sports Center, Xi'an Jiaotong University, Xi'an, Shaanxi, China; ^2^Department of Dermatology, Tianjin Academy of Traditional Chinese Medicine Affiliated Hospital, Tianjin, China; ^3^Department of Human Anatomy, Histology and Embryology, School of Basic Medical Sciences, Xi’an Jiaotong University Health Science Center, Xi’an, Shaanxi, China; ^4^Health Science Center, Xi’an Jiaotong University, Xi’an, Shaanxi, China; ^5^Department of Library, The Second Affiliated Hospital of Xi'an Jiaotong University, Xi'an, Shaanxi, China

**Keywords:** resistance training, aging, exercise, strength, skeletal muscle, muscle strength, bibliometrics, visual analysis

## Abstract

**Purpose:**

Resistance training (RT) can intervene in aging, which can effectively improve trainees’ life. However, unhealthy living habits such as irregular life, obesity and hyperlipidemia, and chronic diseases lead to a significant decline in the energy level of the population, seriously affecting the health of the population. Our research identifies the research hotspots of RT to intervene in aging from the perspective of bibliometrics, predicts research frontiers and development trends, and provides more perspectives for research on aging populations.

**Methods:**

In this study, we used CiteSpace and VOSviewer visualization software to draw the scientific knowledge map of countries/regions, institutions, authors, co-occurrence keywords, and co-cited references of published articles, and explore the Web of Science core collection database all about the RT intervention aging research status, hotspots, frontiers, and development trends of articles on aging.

**Results:**

Among the 760 articles that meet the inclusion criteria, the number of articles published and the frequency of citations have increased steadily in the past 5 years. Judging from the countries/regions, institutions, scholars, and journals that published articles, the ones with the largest numbers are the USA, Univ Estadual Londrina, Cyrino ES, and *Exp Gerontol*. The ones with the highest influence are England, Univ Arkansas Med Sci, Frontera WR, and *Biochem Biophys Rep Co*. The top five co-occurrence keywords of include exercise, strength, resistance training, skeletal muscle, and muscle strength. The research frontier is physical function.

**Conclusion:**

In the field of RT intervention aging research, relevant scholars deserve further in-depth research and exploration. The United States, Brazil, Canada, and other economically developed countries/regions, institutions, and authors have greater influence and productivity. These quantitative research results can provide references for relevant scholars’ follow-up research and government departments to formulate and modify health policies or measures.

## 1. Introduction

Resistance training (RT), also known as isotonic training, isometric training, or isokinetic training, refers to the use of fixed combination equipment or free weight equipment (such as dumbbells, kettlebells, and elastic bands/ropes) to maintain muscle strength weight training at a constant speed can effectively improve the endurance, speed, flexibility, and strength of athletes’ skeletal muscles (1). Human aging is related to genetics, environment, behavior, demographic characteristics, and chronic diseases (2–5). Although the rapid development of social sciences and the significant improvement of economic capacity have reduced disease mortality and extended the life expectancy of the population, the incidence of cardiovascular, metabolic, skin, and other chronic diseases have shown a diversification and rising trend (6). Along with the increase of age, they will also be prone to falls due to the decrease of protein synthesis, which leads to the impairment of muscle contraction and strength even in the elderly population without any disease, the progression of aging can lead to physical disability, reduced mobility or falls as we age (7–12). There is substantial evidence showing that RT increases skeletal muscle mass (SMM) and decreases fat mass in the older population (13, 14). RT can improve the muscle mass and muscle strength of elderly people (15–17) and effectively supplement the health and physical fitness of athletes in aerobic training (18–21). Furthermore, it can be asserted that the changes in muscle strength levels could predict the changes in the levels of cognition in older women (22, 23). The method, action, frequency, speed, and interval time of RT are all related to the training effect (24–27). The prevalence of metabolic syndrome and hypertension increases with age, and aerobic exercise combined with resistance training helps maintain good health and produces important metabolic adaptations in older adults (28, 29). However, this study suggests that load intensity does not seem to determine the RT effect on several obesity-related pro-inflammatory and chemotactic compounds, body fat, IRM, and 6MWT in postmenopausal women (30), and a lifetime of heavy resistance training does not appear to alter left ventricular function compared to age-matched controls (31)， and obesity, hyperlipidemia, and decreased physical fitness caused by unhealthy living habits such as irregular life, excessive calorie intake, and reduced exercise are becoming more and more serious, which is an important impact on the health of middle school students, college students, urban white-collar workers, and the elderly factors (32–36).

At present, the publications related to RT research are growing rapidly, and the content of the research has been subdivided and in-depth, which has played a positive role in the development of the research field of RT intervention of aging. However, it is difficult for relevant personnel to quickly and accurately grasp the research hotspots, frontiers, and development trends in the field of RT intervention aging research, which limits the in-depth development of physical education teaching, RT, and research work to a certain extent. Therefore, this study intends to base itself on the ISI Web of Science (WoS) core collection database, use CiteSpace and VOSviewer visual analysis software to draw the scientific knowledge map of publications related to the field of RT intervention aging research, and combine expert opinions to explore its research. The status quo, hotspots, frontiers, evolution paths, and development trends are expected to provide references for relevant personnel to systematically and scientifically understand the research trends in the field of RT to intervene in aging.

## 2. Materials and methods

### 2.1. Data sources

Log in to the Web of Science database platform (http://apps.web of knowledge.com/), select the core collection database, and use “resistance training” AND “aging “as the subject terms to conduct a full-text search. Refinement strategy: (1) Document Type: Article; (2) Language: English; (3) Time span: from the establishment of the database to the present; the rest are system default parameters. Retrieved: 27 October 2022.

### 2.2. Literature retrieval and preservation

**Figure 1:**
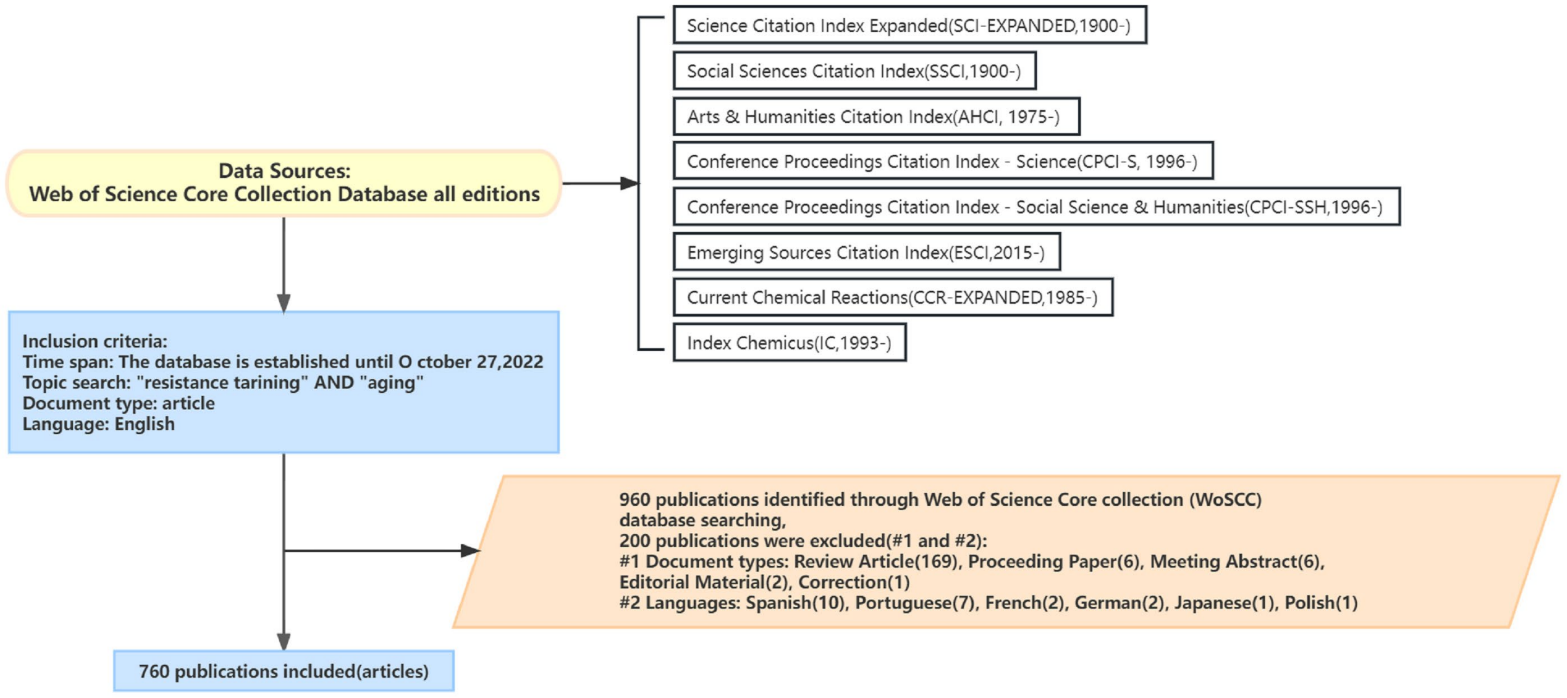
Literature screening process.

Search strategy references (37–39) were developed by two researchers who have received systematic training in evidence-based practice methods to independently screen literature, extract data, and conduct cross-checks. If there is any disagreement, other members of the research team are invited to discuss and judge. For all the literature obtained in the preliminary screening, check the topics one by one to determine whether it is related to resistance training and aging research, and continue to read the abstract for a demonstration before it can be included in this study. If it does not meet the inclusion criteria and is re-argued after reading the full text, it will be excluded. In the end, a total of 760 related publications were obtained, and all selected publications were added to the “Add to Made List,” the data files were downloaded according to Export → plain text file → Full Record and Cited Reference, saved as “plain text” format, and named and saved as “download_xxx,” respectively. The literature screening process is shown in Figure 1.

### 2.3. Data extraction project

The content of data extraction mainly includes literature title, cited references, published author, corresponding author, journal, country, institution, publication time, language, type, keyword, abstract, issue number, and page number of the article.

### 2.4. Descriptive statistical analysis

Taking the collected publications in the field of RT intervention of aging research as the research object, combined with the relevant data on the WoS database search results analysis page, the annual citation frequency and the number of publications were entered into Excel2019 for statistical analysis and drawing trend charts.

### 2.5. Data visualization analysis

We measure the production capacity and influence of countries/regions, institutions, and authors by the number of annually published publications and citations in the subject area, using H-index and centrality in indices and scientific knowledge graphs to assess the academic contributions of countries, institutions, and authors and to predict their future scientific achievements. The quality and impact of journals published are measured according to the *Journal Citation Reports* (2022) Lmpact factor (IF). We used CiteSpace6.1.R3 and/or VOSviewer1.6.17.0 visual analysis software to extract relevant information units, respectively, draw the co-authored maps of countries/regions, institutions, and authors to explore the research status, draw the total number of journals, authors, and references citing clustering/emergence/timeline graphs and journal double graph overlay analysis to explore frontier research content and development trends, and draw co-occurrence keywords and emergent keywords scientific knowledge graphs to explore research hotspots. The method of judging the results can be found in the references (40).

## 3. Result

### 3.1. Bibliometric analysis of publication outputs

**Figure 2:**
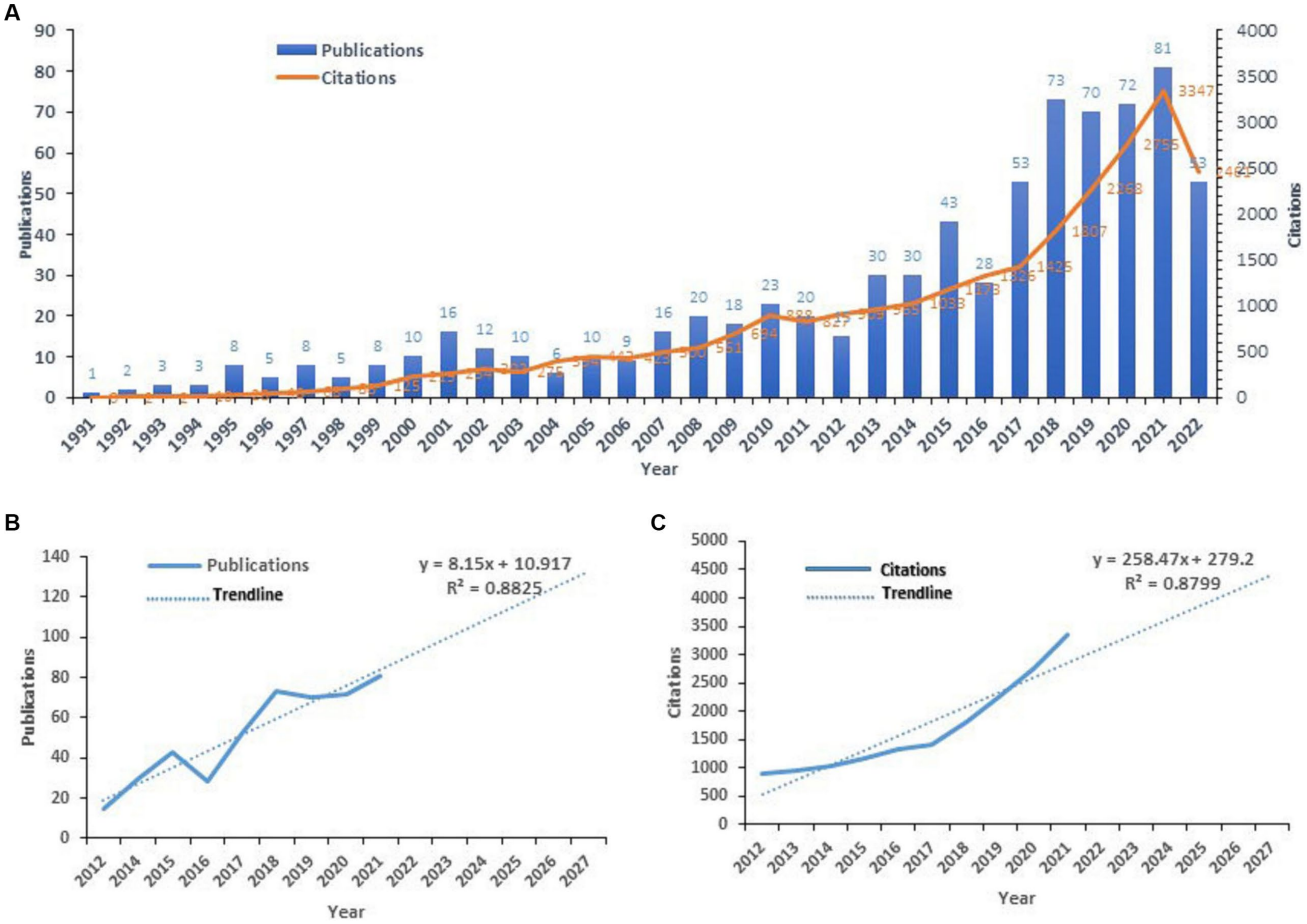
The publication distribution and citation status of relevant publications in the field of resistance training intervention aging research **(A)** and the development trend in the next 5 years **(B,C)**.

Until now, 760 publications related to the field of RT intervention aging research have been cited 25,618 times, with an H-index of 84. There were some fluctuations, but they all showed a good upward trend on the whole (Figure 2A), and the fitting curve spectrum also suggested that there would be a steady upward trend in the next 5 years (Figures 2B,C). This shows that relevant scholars are paying more and more attention to the research field of RT intervention aging.

### 3.2. Research direction and journal analysis

The 760 articles were published in 245 scientific and technological journals, covering 46 research directions in 58 subject areas. The top 10 productive journals are listed in Table 1. From the co-citation map of publications (Supplementary Figure 1), we can see that the journal with the highest total citation frequency is *Med Sci Sport Exer* and the journal with the highest centrality is *Biochem Bioph Res Co*.

**Table 1:** Details of the top 10 journals in the field of resistance training intervention aging research in terms of the number of published papers, 1991–2022.

Rank	Publication Titles	Record Count	Citations	Category Rank	2021IF/Category Quartile	JCR Category	H-index	Country
1	*Exp Gerontol*	53	1,251	26/54	4.253/Q2	Geriatrics and Gerontology	20	England
2	*J Strength Cond Res*	49	1,513	18/88	4.415/Q1	Sport Sciences	20	USA
3	*J Appl Physiol*	39	3,554	27/81	3.881/Q2	Physiology	29	USA
4	*J Aging Phys Activ*	27	554	47/54	2.109/Q4	Geriatrics & Gerontology	16	USA
5	*Med Sci Sport Exer*	23	1978	9/88	6.289/Q1	Sport Sciences	19	USA
6	*Front Physiol*	21	433	20/81	4.755/Q1	Physiology	9	Switzerland
7	*Eur J Appl Physiol*	20	703	32/81	3.346/Q2	Physiology	15	Germany
8	*Int J Sports Med*	18	328	41/88	2.997/Q2	Sport Sciences	10	Germany
9	*Aging Clin Exp Res*	16	421	23/54	4.481/Q2	Geriatrics and Gerontology	11	Italy
10	*Appl Physiol Nutr Me*	14	432	62/90	3.016/Q3	Nutrition and Dietetics	10	canada
11	*J Gerontol A-Biol*	14	1,139	13/54	6.591/Q1	Geriatrics and Gerontology	10	USA

### 3.3. Country/region analysis

**Figure 3:**
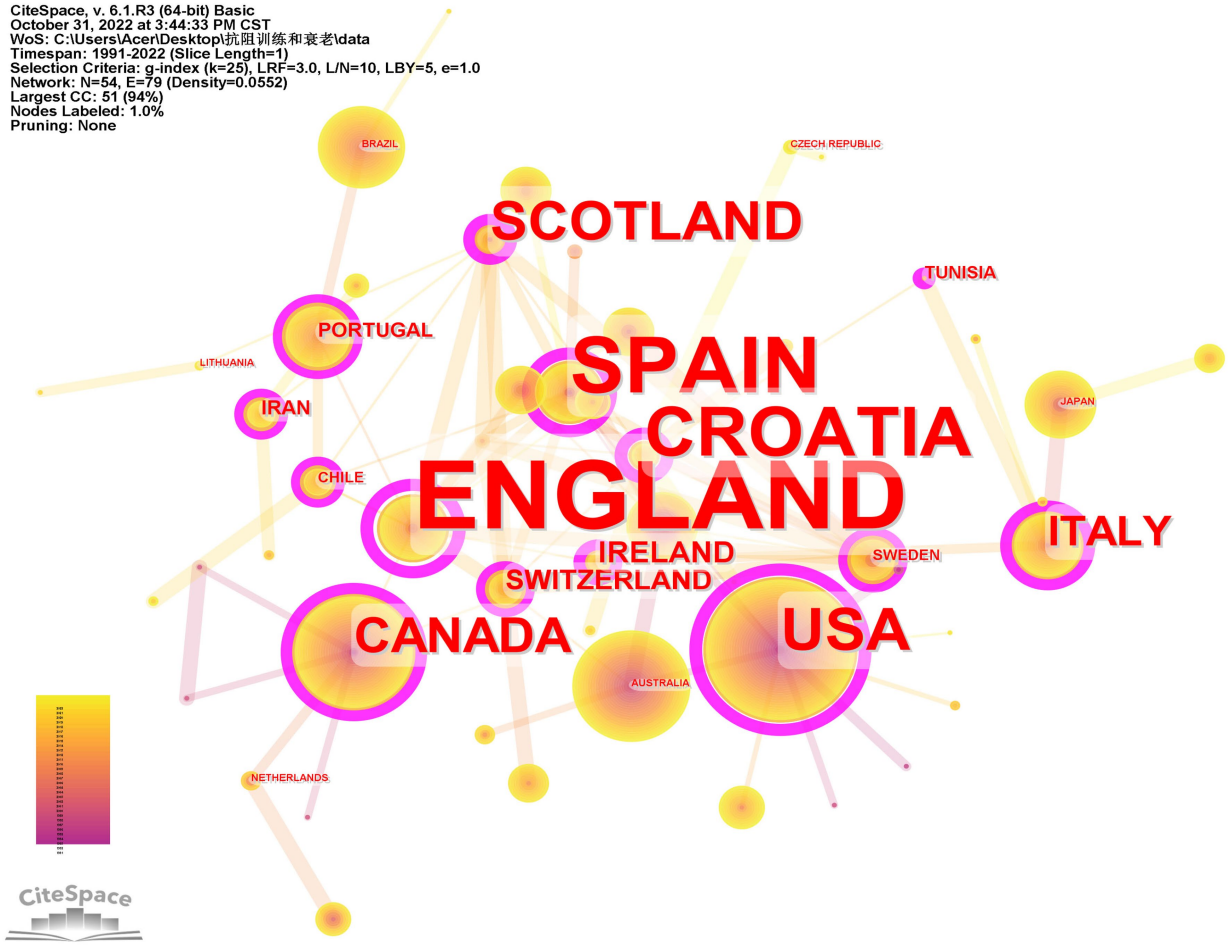
National/regional scientific research cooperation map of publications and journals in the field of resistance training intervention aging research.

A total of 48 countries/regions have participated in research in the field of RT intervention aging research. The top three countries/regions in the number of published articles are the USA, Brazil, and Canada, and the top three countries/regions in terms of centrality are England, Spain, and Croatia (Supplementary Table 1). The countries/regions with purple outer circle nodes in Figure 3 play an important “bridge” role in scientific research cooperation in this field. Until now, Italy, Japan, Australia, Scotland, Malaysia, Peoples R China, China Taiwan, and Slovenia’s research is the most popular, and they have extensive scientific research co-authorship.

### 3.4. Institutions analysis

**Figure 4:**
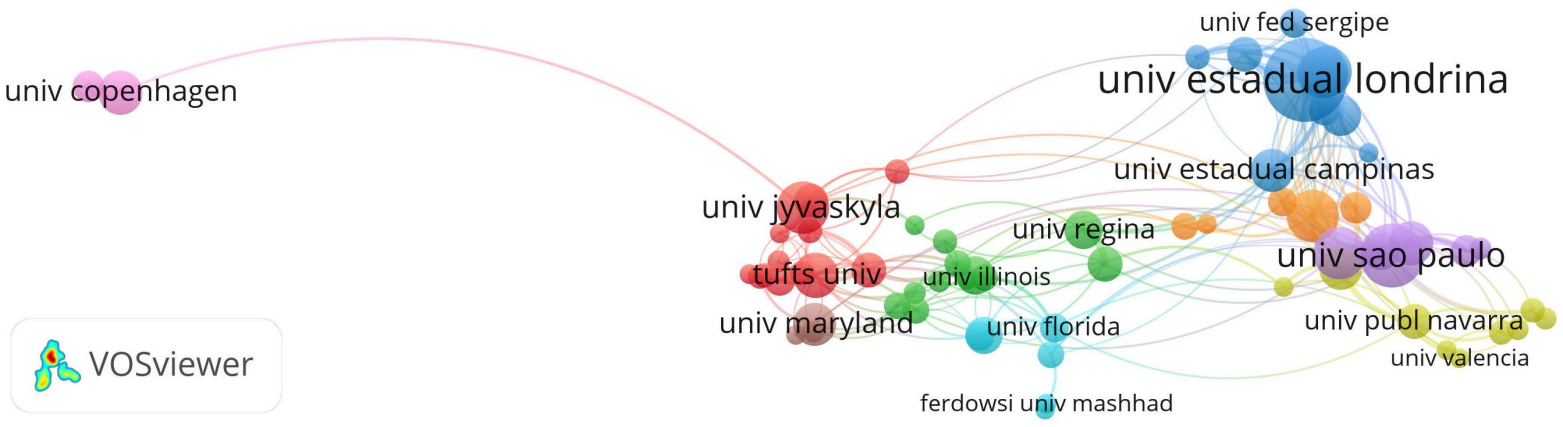
The map of scientific research cooperation relationship between the authored institutions of articles related to resistance training intervention aging research (66 institutions co-authored ≥6 articles, connecting 190 lines, forming 7 clusters).

This group of articles has a total of 1,040 signature institutions. According to Price’s law, there are 66 core publication institutions with signatures ≥6 articles. The top three institutions in terms of productivity are Univ Estadual Londrina, Univ São Paulo, and the City University of New York. The top most influential institution is Univ Arkansas Med Sci (Supplementary Table 2). Figure 4 is a map of the co-author relationship of the authoring institutions of articles in the field of RT intervention of aging research.

### 3.5. Author and co-cited author analysis

**Figure 5:**
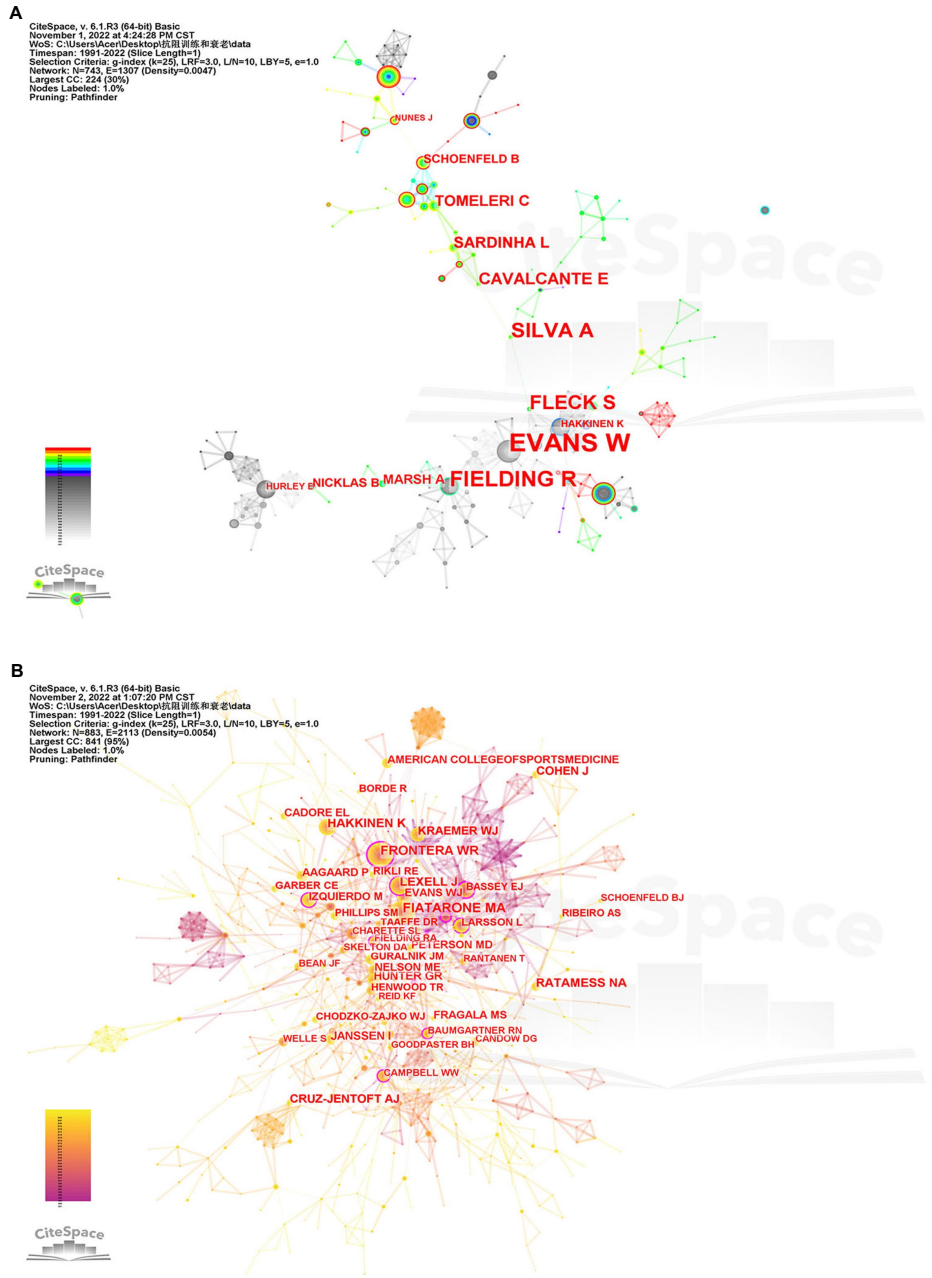
Co-authorship map in the field of resistance training intervention aging research (**A**, co-author ≥5, with 260 connections, forming six clusters), and co-citation relationship map of authors **(B)** of publications in the field of resistance training intervention aging research.

There are 3,187 signed authors. The top three authors in the number of published articles are Cyrino ES, Ribeiro AS, and Schoenfeld BJ. The top three authors in betweenness centrality are Evans WJ, Fielding R, and Silva A. It can be seen from Figure 5A that this research field still lacks “key authors” who lead the development direction of the discipline across regions/teams.

Figure 5B is the relationship map of co-cited authors of publications in the field of RT intervention aging research, with a total of 14,718 citing authors, showing that the core author team is closely connected, and a few core authors participate in cross-team and cross-regional collaboration study. The top three authors with the most times cited are Fiatarone MA, Frontera WR, and Häkkinen K, and the top three authors of betweenness centrality are Frontera WR, Aniansson A, and Bassey EJ. They have a high academic orientation that represents the hot research content in the subject field and has great potential for scientific research cooperation.

### 3.6. Keyword analysis

**Figure 6:**
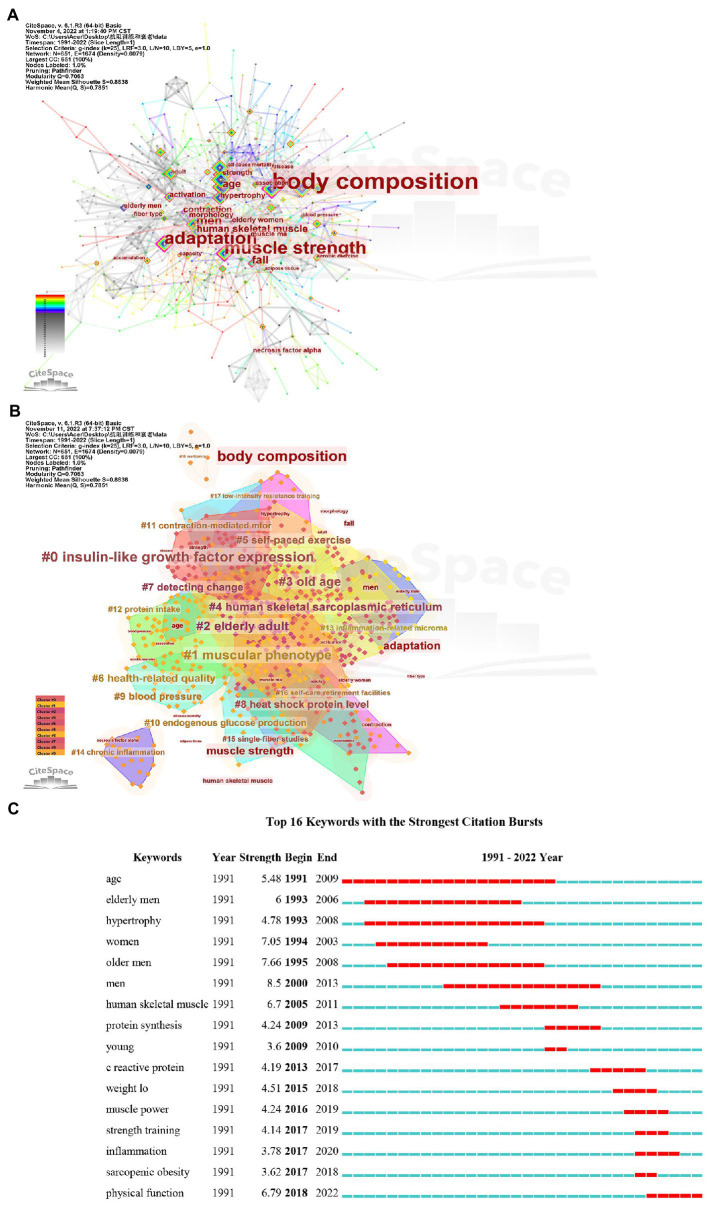
Co-occurrence keyword map **(A)**, co-occurrence keyword cluster map **(B)**, and bursts of co-occurrence keyword map (**C**, The red line segment represents the time when the keyword emerges. The larger the strength value, the larger its burst strength section) of articles in the field of resistance training intervention aging research.

Figure 6A shows the co-occurrence keyword map of publications related to RT intervention aging research. There are a total of 15,236 co-occurrence keywords. According to Price’s law, we judge that 51 core keywords can reflect the research hotspots in this subject field (Co-occurrence frequency ≥ 93 times), among which the top five keywords of research popularity include exercise (285 times), strength (211 times), resistance training (191 times), skeleton muscle (150 times), and muscle strength (110 times). The top five most influential keywords are body composition, muscle strength, adaptation, men, and fall. According to Figure 6B, we speculate that these keywords constitute 19 visible clusters, and the order of the cluster ID numbers from small to large reflects the evolution path of the research content involved in the keywords in the clusters from near to far (Supplementary Table 3). According to Figure 6C, we speculate that the current research frontier in the field of RT intervention aging research is physical function.

### 3.7. References analysis

**Figure 7:**
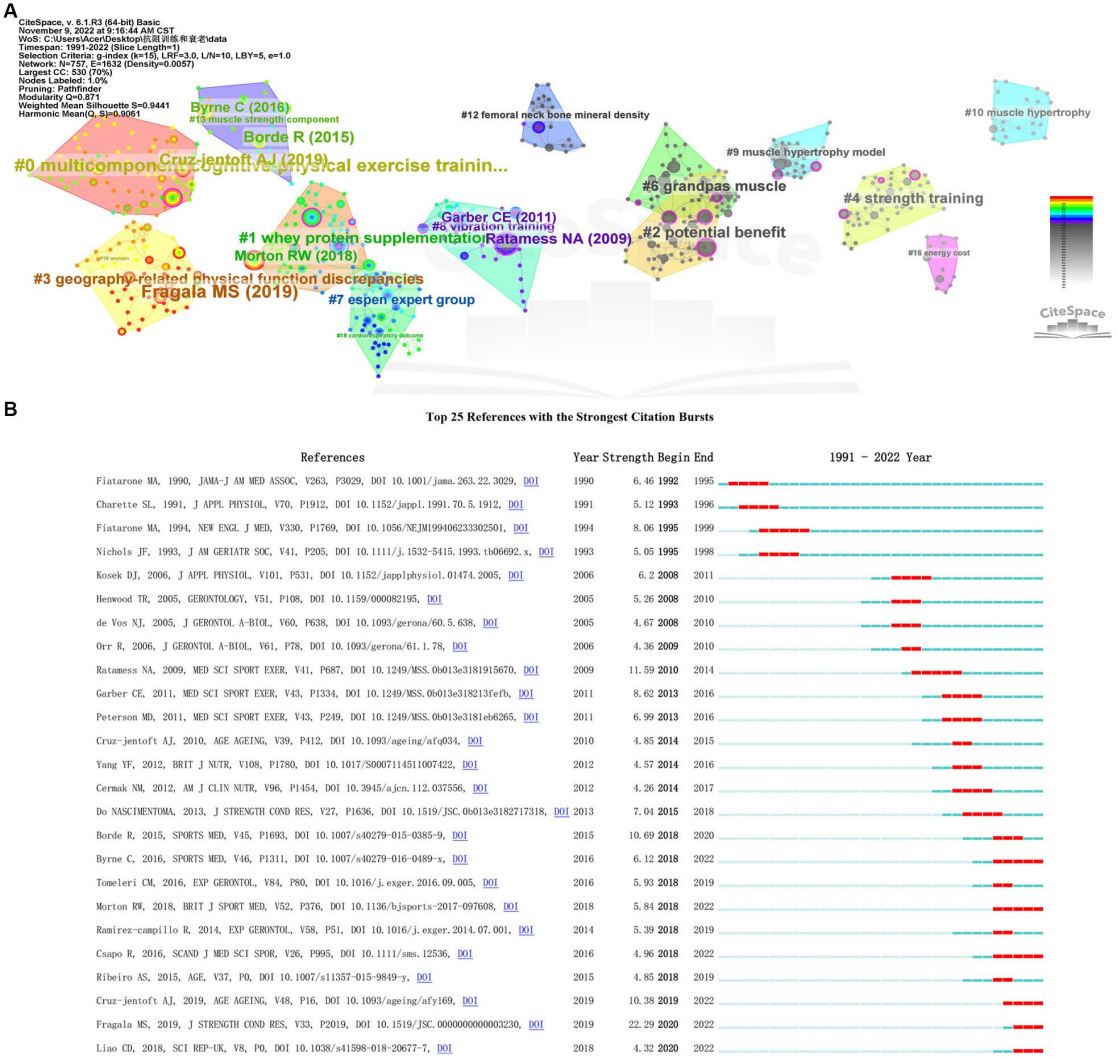
Clustering map of co-cited references **(A)** and bursts of co-cited references **(B)** in publications related to resistance training intervention aging research (The red line represents the emergence time of the references. The references with larger burst values have higher research content).

The total number of citations of this group of articles in the WoS database is 25,618 times. Among the five most cited articles, Fragala et al. (41) ranked first in *J Strength Cond Res* in 2019 (319 times) and Dalle et al. (42) ranked second in 2017 in *Front Physiol* (250 times, Supplementary Table 4). Among all 21,975 cited references in the local dataset, 435 references with a total of ≥8 citations determined by Price’s law were considered core co-cited references in the field. There are 15 visible clusters in the clustering map of co-cited references (Figure 7A), and the ordering of ID numbers from small to large reflects the development of the research content of publications related to RT intervention of aging from near to far in time path (Supplementary Tables 5–7). Figure 7B shows the detection results of the emergence analysis of co-cited references. It is suggested that the research contents of Byrne C, Morton RW, Csapo R, Cruz-jentoft AJ, Fragala MS, and Liao CD represent the research frontier in the field of RT intervention aging, especially Fragala et al. (41) published in *J* *Strength Cond Res* had the highest burst intensity (22.29), which again shows that RT has a good effect to intervene in aging and prolonging the life expectancy of the elderly people.

## 4. Discussions

The results of this study showed that 3,187 authors from 1,040 institutions in 48 countries participated in the 760 articles that met the inclusion criteria, and their articles were published in 245 scientific journals. It covers 46 research directions in 58 subject areas. The main findings of the survey are as follows:

(1) In the past 5 years, the research on RT intervention aging has received more and more attention from scholars (402 articles, accounting for 52.89%).
(2) The United States has the largest number of publications, but publications published in England had the highest impact. In addition, the top nine countries/regions are all located in economically developed regions in Europe and America.
(3) Among the top 10 institutions with the number of publications (305, accounting for 40.13%), Brazil and USA research institutions each account for half. Brazil’s Univ Estadual Londrina has the largest number of publications.
(4) Among the top 10 authors in the number of published publications, Brazilian authors accounted for five, among which Cyrino ES of Univ Estadual Londrina (Brazil) had the highest productivity.

Relevant scholars should focus on the research results of countries/regions, institutions, and authors with high productivity and influence. This is one of the effective ways to analyze and mine current research hotspots and cutting-edge content, and it is possible to explore an efficient research path to publish more high-quality research results in recognized and influential authoritative journals in future.

(5) RT is one of the important contents of aging intervention/delay: ①The authors with the highest research interest include Fiatarone MA, Frontera WR, and Häkkinen K. Their representative studies believe that progressive RT is an effective antidepressant in depressed elderly people, while also improving strength, morale, and quality of life (43); their latest research suggests that RT induces great differences in muscle strength and mass responses between male and female participants of different ages (44). ②The most influential authors are Frontera WR, Aniansson A, and Bassey EJ. Their representative studies pointed out that the short-term high-speed strength training and traditional slow progressive RT intervention functionally limited the lower limb neuromuscular strength of elderly people. The effects of training are comparable, but the former can promote muscle mass improvement by changing neuromuscular adaptations (45). ③Currently, Ribeiro AS, Borde R, Cohen J, Cruz- Jentoft AJ, Liao CD, and Fragala MS are the most active authors, and the publication of Fragala MS [title: Resistance Training for Older Adults: Position Statement From the National Strength and Conditioning Association (41)] was the most explosive, and the publications of Fiatarone MA had the highest citation frequency (the research enthusiasm for the effective treatment of elderly depression with progressive resistance training was the highest) (43). A representative study of their studies found that core strengthening training can effectively improve the comprehensive balance ability of elderly people (46). Their latest study found that the application of progressive RT (PRT) can effectively counteract the decline in muscle strength and function in elderly people (47). All of the above suggests that the research content in the field of RT intervention aging has good continuity.
(6) Although RT has long been recognized as an important component of aging management, clinicians rarely prescribe RT intervention in aging. Through the scientific knowledge map of co-occurring keywords, we found that the most commonly used keywords in 760 publications are exercise, strength, RT, skeletal muscle, muscle strength, body composition, physical activity, and adult, and the most influential keywords are body composition, muscle strength, adaptation, fall, human skeletal muscle, and contraction. The publications with the highest research interest involve the national position statement on RT for elderly people (41). Protein supplementation after RT [preferably dominant whey protein, ≥ (1.0 ~ 1.2) g/(kg·d)] has good potential for anti-inflammatory treatment (42, 48). The European Society for Clinical Nutrition and Metabolism (ESPEN) also clearly stated that all elderly people should carry out daily physical activities or exercise (RT) for as long as possible (12). The latest research pointed out that the improvement of knee strength of elderly resistance trainers was significantly better than that of elderly runners (49), especially for 10 weeks of sprint intervals, while performing RT is particularly important for effectively improving the body composition and muscle strength of inactive elderly women (16), which once again confirmed that 16 weeks of aerobic exercise combined with RT can effectively improve metabolic syndrome in elderly women and harm to the body (28).
(7) We speculate that the current research frontier in the field of RT intervention aging is physical function. Representative studies found that intensive RT can improve the physical function of elderly people and prevent preclinical disability (50). The latest research found that after the elderly people received eccentric overload flywheel training, their physical function, balance, and mobility were enhanced, and their muscle thickness and muscle mass were also significantly improved compared with those before training (51).

Co-cited reference analysis helps us analyze disciplines, research frontiers, and global trends in fields by exploring “key references.” In the clustering map of co-cited references, the ordering of ID numbers from small to large reflects the development of the research content of publications related to RT intervention aging from near to far in the time path (The top five most important clusters are #0 multicomponent cognitive-physical exercise training, #1 whey protein supplementation, #2 potential benefit, #3 geography-related physical function discrepancies, and #4 strength training; Supplementary Table 5). From the perspective of the influence of co-cited references, the top three are Ratamess NA, Garber CE, and Do Nascimentoma MA. It is necessary and important to formulate a step-by-step RT program research content in advance with the trainer’s goal, physical fitness, and training status varying from person to person (52). It also advocates for healthy adults of all ages or those with certain chronic diseases or disabilities and adults provided with individualized exercise prescriptions (53) (Supplementary Table 6). In addition, from the perspective of the contribution of co-cited references, the top three are the studies of Fragala MS, Cruz-jentoft AJ, and Borde R. Their latest study pointed out that it is necessary to prescribe RT and prevent it for elderly people without chronic diseases (41), and found that the time, intensity, tension time, and interval rest of RT play an important role in the improvement of muscle strength and body shape of healthy elderly people (54), but restricted and optimal RT dose–response relationships in frail older populations require further investigation (Supplementary Table 7).

Finally, we also explore the research frontiers of RT of the intervention in aging through highly cited publications, which may have future research hotspots. Their research contents, in order, are as follows: emphasizing that RT is effective in intervention of aging (12), pointing out that classic interventions to combat age-related muscle wasting mainly focus on RT and/or protein supplementation (41), and whey protein is the best (42), especially the daily protein diet and long-term adherence to RT and aerobic exercise are very necessary for the elderly people (48). Both ailments and diseases (wasting and cachexia) need to be treated with swallowing muscle RT and nutritional intervention (55) (Supplementary Table 4).

In summary, the author believes that RT has a good application prospect in the intervention aging, and it is worthy of reference for clinicians.

## 5. Conclusion

Our findings suggest that the field of RT intervention/delay in aging research remains promising. Countries, institutions, and authors in economically developed regions such as the United States, Brazil, and the United Kingdom have great influence and productivity in this field. Institutions and scholars in economically underdeveloped regions such as Asia and Africa can deeply explore horizontal and vertical scientific research cooperation with the potential. “Physical function” is probably the newest research frontier right now. Overall, this study provides a scientific perspective on RT as a intervention of aging research and can serve as a reference for those involved.

## 6. Advantages and limitations

This study is the first in the world to use bibliometric methods to investigate the research hotspots and global development trends of publications in the field of RT intervention of aging research. Our research provides a new historical perspective on the development of the subject area, objectively analyzing and exploring the content of different research aspects of publications in this group. However, this study still has some limitations. First, only the publications of the core collection database of WoS were searched, and the publications published by Scopus, CNKI, and Wanfang databases were not involved. Second, we excluded non-English publications, a limitation that may have introduced publication bias. Third， although the research content of this group is updated more and faster, research on physical activity/exercise strategies that are not conducive to the physical function of healthy people and elderly people is relatively rare.

## Data availability statement

The original contributions presented in the study are included in the article/Supplementary materials, further inquiries can be directed to the corresponding authors.

## Author contributions

JM, LZ, and YZ were responsible for data analysis and the initial draft. JZ and JM revised the second draft according to feedback from PY, WS, and ZW. JZ, PY, WS, and ZW assisted in a literature search. LZ and YZ contributed to the study’s conception and design. All authors contributed to the article and approved the submitted version.

## Funding

This study was supported by the Key Research and Development Program Project of Shaanxi Province (Research Project Number: 2019SF-150).

## Conflict of interest

The authors declare that this study was performed without any commercial or financial relationships that could be construed as potential conflicts of interest.

## Publisher’s note

All claims expressed in this article are solely those of the authors and do not necessarily represent those of their affiliated organizations, or those of the publisher, the editors and the reviewers. Any product that may be evaluated in this article, or claim that may be made by its manufacturer, is not guaranteed or endorsed by the publisher.

## Supplementary material

The Supplementary material for this article can be found online at: https://www.frontiersin.org/articles/10.3389/fpubh.2023.1133972/full#supplementary-material
