# Supplementary material for: Research hotspots and new trends in the impact of resistance training on aging, bibliometric and visual analysis based on CiteSpace and VOSviewer
Source: Front Public Health. 2023 Jun 2;11:1133972. doi: 10.3389/fpubh.2023.1133972 (PMC10275612; doi:10.3389/fpubh.2023.1133972)
Supplement: Supplementary file 1 [file Table_1.pdf]

Supplementary Table 1 Details of the top 10 countries/regions in the field of resistance training to inhibit aging research in the number of published papers and centrality, 1991-2022

Rank	Country/region	Publications	Centrality	Citations	H-index	Year	Country/region	Centrality	Publications	Citations	H-index	Year
1	USA	338	0.41	15429	69	1993	England	0.66	25	1202	15	2004
2	Brazil	195	0.07	3407	30	2002	Spain	0.55	40	2382	18	1998
3	Canada	75	0.34	2763	69	1993	Croatia	0.47	6	793	5	2014
4	Australia	51	0.08	2399	21	1997	USA	0.41	325	15429	69	1993
5	Spain	41	0.55	2382	18	1998	Scotland	0.38	7	908	6	2003
6	Portugal	37	0.14	927	16	2009	Canada	0.34	73	2763	69	1993
7	Italy	31	0.29	1335	13	2002	Italy	0.29	30	1335	13	2002
8	Denmark	26	0.02	799	13	2007	Ireland	0.20	5	141	4	2013
9	England	26	0.66	1202	15	2004	Switzerland	0.19	8	977	7	2009
10	Iran	25	0.14	145	8	2013	Portugal	0.14	35	927	16	2009
