# Supplementary material for: Research hotspots and new trends in the impact of resistance training on aging, bibliometric and visual analysis based on CiteSpace and VOSviewer
Source: Front Public Health. 2023 Jun 2;11:1133972. doi: 10.3389/fpubh.2023.1133972 (PMC10275612; doi:10.3389/fpubh.2023.1133972)
Supplement: Supplementary file 2 [file Table_2.pdf]

Supplementary Table 2 Details of the top 10 institutions in the field of resistance training to inhibit aging research in the number of published papers and centrality, 1991-2022

Rank	Institution	Publica options	Country/ region	Institution	Centrality	Country/ region
1	Londrina State University	49	Brazil	Univ Arkansas Med Sci	0.19	USA
2	Univ Sao Paulo	31	Brazil	Tufts Univ	0.17	USA
3	City University of New York (CUNY)	29	USA	Campinas State University	0.14	Brazil
4	Us Department of Veterans Affairs	27	USA	Brandon Univ	0.14	Canada
5	Univ Brasilia	26	Brazil	Auburn Univ	0.13	USA
6	State University System of Florida	25	USA	Univ São Paulo	0.12	Brazil
7	Veterans Health Administration(VHA)	25	USA	Univ Sydney	0.12	Australia
8	Geriatric Research Education Clinical Center	24	USA	Univ Illinois	0.11	USA
9	Tufts Univ(USDA)	23	USA	Univ British Columbia	0.11	Canada
10	Univ Fed Santa Catarina	23	Brazil	Univ Catolica Brasilia	0.10	Brazil
11	Univ Norte Parana	23	Brazil	Penn State Univ	0.10	USA
