# Supplementary material for: Research hotspots and new trends in the impact of resistance training on aging, bibliometric and visual analysis based on CiteSpace and VOSviewer
Source: Front Public Health. 2023 Jun 2;11:1133972. doi: 10.3389/fpubh.2023.1133972 (PMC10275612; doi:10.3389/fpubh.2023.1133972)
Supplement: Supplementary file 3 [file Table_3.pdf]

Supplementary Table 3 Basic characteristics of the top 10 main clusters in the keyword clustering diagram of related papers in the field of resistance training to inhibit aging research,1991-2022

I D	size	Silhouette	Average Year	Label (LLR)	The most cited members in this cluster are:
# 0	70	0.927	2003	insulin-like growth factor expression	exercise（285）, strength（211）, body composition（102）
#1	60	0.753	2013	muscular phenotype	physical activity（100）, women（84）, power(52)
#2	55	0.91	1999	elderly adult	resistance training(191),skeletal muscle(150),muscle strength(110)
#3	54	0.879	2005	old age	adult(100),hypertrophy(67),muscle(29)
#4	45	0.936	2000	human skeletal sarcoplasmic reticulum	adaptation(56),sarcopenia(46),elderly men(20)
#5	39	0.848	2005	self-paced exercise	fitness(44),health(41),balance(28)
#6	39	0.858	2014	health-related quality	quality of life(35),aerobic exercise(22),mortality(22)
#7	38	0.873	2002	detecting change	men(98),muscle ma(32),muscle hypertrophy(18)
#8	37	0.902	2006	heat shock protein level	muscle power(23),expression(21),validation(7)
#9	35	0.905	2011	blood pressure	older adult(87),functional capacity(14),muscular strength(13)
