# Supplementary material for: Research hotspots and new trends in the impact of resistance training on aging, bibliometric and visual analysis based on CiteSpace and VOSviewer
Source: Front Public Health. 2023 Jun 2;11:1133972. doi: 10.3389/fpubh.2023.1133972 (PMC10275612; doi:10.3389/fpubh.2023.1133972)
Supplement: Supplementary file 4 [file Table_4.pdf]

Supplementary Table 4 Basic characteristics of the highly cited related papers in the field of resistance training to inhibit aging research, 1991-2022

Rank	Publication Titles	Journal	Citations	DOI	Year	Author
1	Resistance Training for Older Adults: Position Statement From the National Strength and Conditioning Association	*J Strength Cond Res*	319	10.1519/JSC.0000000000003230	2019	Fragala MS, et al
2	The Role of Inflammation in Age-Related Sarcopenia	*Front Physiol*	250	10.3389/fphys.2017.01045	2017	Dalle S, et al
3	Supplemental Protein in Support of Muscle Mass and Health: Advantage Whey	*J Food Sci*	164	10.1111/1750-3841.12802	2015	Devries MC, et al
4	Protein intake and exercise for optimal muscle function with aging: Recommendations from the ESPEN Expert Group	*Clin Nutr*	737	10.1016/j.clnu.2014.04.007	2014	Deutz NEP, et al
5	Sarcopenia and dysphagia: Position paper by four professional organizations	*Geriatr Gerontol Int*	143	10.1111/ggi.13591	2019	Fujishima I, et al
