# Supplementary material for: Research hotspots and new trends in the impact of resistance training on aging, bibliometric and visual analysis based on CiteSpace and VOSviewer
Source: Front Public Health. 2023 Jun 2;11:1133972. doi: 10.3389/fpubh.2023.1133972 (PMC10275612; doi:10.3389/fpubh.2023.1133972)
Supplement: Supplementary file 5 [file Table_5.pdf]

Supplementary Table 5 Basic characteristics of the 10 main clusters in the clustering map of co-cited references of related papers in the field of resistance training to inhibit aging research, 1991-2022

| I D | size | Silhouette | Average Year | Label (LLR) | The major citing article of the cluster is: |
|---|---|---|---|---|---|
| # 0 | 74 | 0.922 | 2019 | multicomponent cognitive-physical exercise training | Fragala MS, et al. Resistance training for older adults: position statement from the national strength and conditioning association. |
| #1 | 55 | 0.929 | 2016 | whey protein supplementation | Nabuco HCG, et al. Effects of protein intake beyond habitual intakes associated with resistance training on metabolic syndrome-related parameters, isokinetic strength, and body composition in older women. |
| #2 | 52 | 0.919 | 2008 | potential benefit | Tarnopolsky MA, et al. The potential benefits of creatine and conjugated linoleic acid as adjuncts to resistance training in older adults. |
| #3 | 51 | 0.947 | 2021 | geography-related physical function discrepancies | Valenzuela PL, et al. Physical exercise in the oldest old. |
| #4 | 48 | 0.921 | 2002 | strength training | Porter MM. The Effects of Strength Training on Sarcopenia. |
| #6 | 47 | 0.940 | 2008 | grandpas muscle | Phillips SM. Resistance exercise: good for more than just grandma and grandpa's muscles. |
| #7 | 42 | 0.944 | 2014 | espen expert group | Deutz, NEP, et al. Protein intake and exercise for optimal muscle function with aging: recommendations from the espen expert group. |
| #8 | 35 | 0.915 | 2013 | vibration training | Kennis E, et al. Effects of fitness and vibration training on muscle quality: a 1-year postintervention follow-up in older men. |
| #9 | 28 | 0.994 | 2004 | muscle hypertrophy model | Always SE, et al. Muscle hypertrophy models: applications for research on aging. |
| # 10 | 26 | 1.000 | 2000 | muscle hypertrophy | Roubenoff R. Sarcopenia and its implications for the elderly. |
