# Supplementary material for: Research hotspots and new trends in the impact of resistance training on aging, bibliometric and visual analysis based on CiteSpace and VOSviewer
Source: Front Public Health. 2023 Jun 2;11:1133972. doi: 10.3389/fpubh.2023.1133972 (PMC10275612; doi:10.3389/fpubh.2023.1133972)
Supplement: Supplementary file 6 [file Table_6.pdf]

Supplementary table 6 Basic characteristics of the top 10 co-cited references of related papers in the field of resistance training to inhibit aging research, 1991-2022

Rank	author	Co-cited/frequency (Centrality)	year	Co-cited references
1	Fragala MS, et al.	45 (0)	2019	Resistance Training for Older Adults: Position Statement From the National Strength and Conditioning Association.
2	Cruz-jentoft AJ, et al.	26（0.06）	2019	Sarcopenia: revised European consensus on definition and diagnosis.
3	Borde R, et al.	23（0.03）	2015	Dose-Response Relationships of Resistance Training in Healthy Old Adults: A Systematic Review and Meta-Analysis.
4	Ratamess NA, et al	21（0.49）	2009	American College of Sports Medicine position stand. Progression models in resistance training for healthy adults
5	Morton RW, et al.	18（0.04）	2018	A systematic review, meta-analysis and meta-regression of the effect of protein supplementation on resistance training-induced gains in muscle mass and strength in healthy adults.
6	Byrne C, et al.	16（0.01）	2016	Ageing, Muscle Power and Physical Function: A Systematic Review and Implications for Pragmatic Training Interventions.
7	Garber CE, et al.	16（0.41）	2011	American College of Sports Medicine position stand. Quantity and quality of exercise for developing and maintaining cardiorespiratory, musculoskeletal, and neuromotor fitness in apparently healthy adults: guidance for prescribing exercise.
8	Do Nascimentoma MA, et al	14(0.30）	2013	Familiarization and reliability of one repetition maximum strength testing in older women
9	Fiatarone MA, et al.	13（0.01）	1994	Exercise training and nutritional supplementation for physical frailty in very elderly people.
10	Peterson MD, et al.	13（0.15）	2011	Influence of resistance exercise on lean body mass in aging adults: a meta-analysis.
11	Csapo R, et al	13（0.23）	2016	Effects of resistance training with moderate vs heavy loads on muscle mass and strength in the elderly: A meta-analysis.
