# Supplementary material for: Research hotspots and new trends in the impact of resistance training on aging, bibliometric and visual analysis based on CiteSpace and VOSviewer
Source: Front Public Health. 2023 Jun 2;11:1133972. doi: 10.3389/fpubh.2023.1133972 (PMC10275612; doi:10.3389/fpubh.2023.1133972)
Supplement: Supplementary file 7 [file Table_7.pdf]

Supplementary table 7 Basic characteristics of the top 10 most influential co-cited references of related papers in the field of resistance training to inhibit aging research, 1991-2022

Rank	author	Centrality/(Co-cited frequency)	year	Co-cited references
1	Ratamess NA, et al	0.49（21）	2009	American College of Sports Medicine position stand. Progression models in resistance training for healthy adults
2	Garber CE, et al	0.41（16）	2011	American College of Sports Medicine position stand. Quantity and quality of exercise for developing and maintaining cardiorespiratory, musculoskeletal, and neuromotor fitness in apparently healthy adults: guidance for prescribing exercise.
3	Do Nascimentoma MA, et al	0.30（14）	2013	Familiarization and reliability of one repetition maximum strength testing in older women
4	Kalapotharakos VI, et al	0.26（6）	2004	The effects of high- and moderate-resistance training on muscle function in the elderly
5	Hurley BF, et al	0.25（2）	2011	Strength training as a countermeasure to aging muscle and chronic disease.
6	Kosek DJ, et al	0.24（11）	2006	Efficacy of 3 days/wk resistance training on myofiber hypertrophy and myogenic mechanisms in young vs. older adults.
7	Csapo R, et al	0.23（13）	2016	Effects of resistance training with moderate vs heavy loads on muscle mass and strength in the elderly: A meta-analysis.
8	Verdijk LB, et al	0.23（3）	2009	Skeletal muscle hypertrophy following resistance training is accompanied by a fiber type-specific increase in satellite cell content in elderly men.
9	Häkkinen K, et al	0.17（4）	2001	Changes in electromyographic activity, muscle fibre and force production characteristics during heavy resistance/power strength training in middle-aged and older men and women
10	Bamman MM, et al	0.17（3）	2004	Myogenic protein expression before and after resistance loading in 26- and 64-yr-old men and women.
