# Supplementary figures and images for: Research hotspots and new trends in the impact of resistance training on aging, bibliometric and visual analysis based on CiteSpace and VOSviewer
Source: Front Public Health. 2023 Jun 2;11:1133972. doi: 10.3389/fpubh.2023.1133972 (PMC10275612; doi:10.3389/fpubh.2023.1133972)

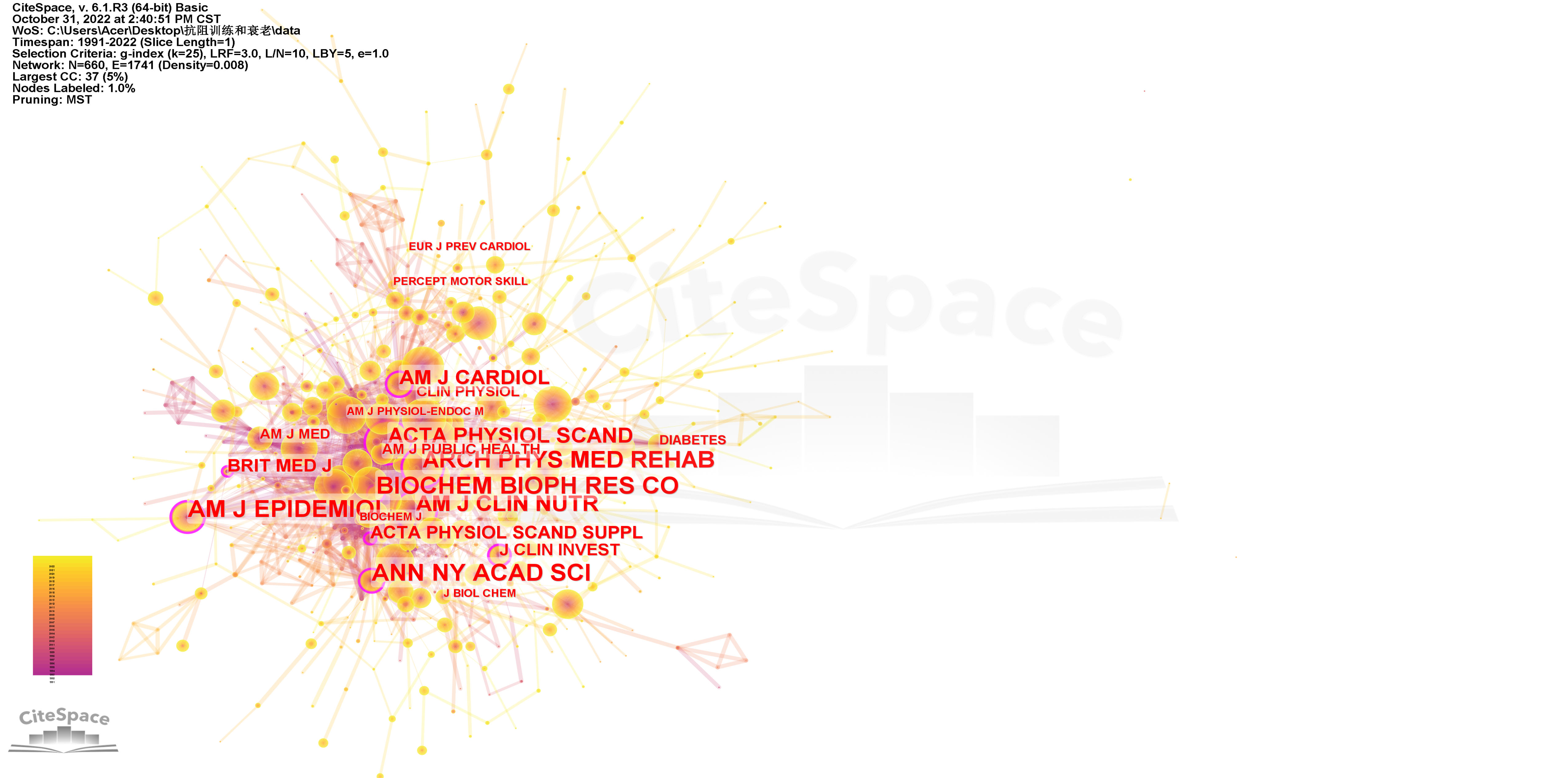

Supplement: Supplementary file 8 [file Image_1.JPEG]
